# Editorial: Cardiovascular Disease and Diabetes

**DOI:** 10.3389/fendo.2019.00314

**Published:** 2019-05-16

**Authors:** Gaetano Santulli

**Affiliations:** ^1^Department of Medicine and Molecular Pharmacology, The Fleischer Institute for Diabetes and Metabolism, The Wilf Family Cardiovascular Research Institute, The Einstein-Mount Sinai Diabetes Research Center, Albert Einstein College of Medicine, New York, NY, United States; ^2^Department of Advanced Biomedical Sciences, “Federico II” University, Naples, Italy

**Keywords:** diabetes mellitus, endocrinology, microRNA, HFpEF (heart failure with preserved ejection fraction), obesity, metabolic syndrome, EPC — endothelial progenitor cells, human induced pluripotent stem cell (iPSC)

People with diabetes mellitus (DM) have a higher-than-average risk of having a heart attack or stroke (1, 2). In fact, DM represents a crucial risk factor for cardiovascular disease (3–5). However, the molecular mechanisms underlying the relationship between DM and cardiovascular disorders are not fully understood; therefore, successful attempts at designing rational interventions remain limited. Nonetheless, recent advances have opened numerous areas of investigation exploring this rapidly evolving research field (6–9), also showing the other side of the coin, i.e., how cardiovascular disease can affect insulin release and glucose homeostasis (10). The present Research Topic aims to present some of the more relevant and recent acquisitions on the molecular mechanisms linking DM and cardiovascular disease, maintaining a focus on the actual translatability in clinical practice.

De Rosa et al., from Magna Graecia University, elegantly illustrated fundamental genetic and epigenetic mechanisms linking cardiovascular disease and DM; similarly, Pordzik et al. identified the functional role of specific platelet-related microRNAs in the pathophysiology of cardiovascular events in high-risk populations, including diabetic patients.

Soares Felicio et al. demonstrated an association between reduced levels of Vitamin D and the presence and severity of diabetic kidney disease in type 1 DM (T1DM); the molecular mechanisms underlying diabetic nephropathy have been also explored by Zou et al. in streptozotocin-induced DM. Arcangeli et al. found a significant association between the number of circulating endothelial progenitor cells (cEPCs) and the age and duration of the disease in T1DM patients: indeed, young T1DM patients have significantly higher levels of cEPCs compared to adult T1DM patients; of note, such difference is also maintained when the disease lasts for more than 10 years. The Authors propose that maintaining a high number of cEPCs, possibly through an efficient glycemic control, would contribute to contain the cardiovascular burden in T1DM. Notably, *in vitro* experiments performed by Lin et al. at New York University have shown how to ameliorate purification and maturation of human induced pluripotent stem cell (iPSC)-derived cardiomyocytes through means of culture in glucose-depleted medium supplemented with fatty acids (oleic acid and linoleic acid) and 3,3′,5-triiodo-L-thyronine (T3).

Applying comprehensive analyses based on imaging and molecular biology, Infante et al. revealed a greater severity of coronary artery disease in type 2 diabetes (T2DM) patients compared to non-diabetic individuals; equally important, van Bussel et al. from Maastricht University Medical Center, highlighted the actual advantages of multiparametric neuroimaging in the clinical evaluation of cognitive decline in T2DM.

The studies performed by Orosz et al. in subjects with impaired glucose tolerance, a prediabetic condition, have shown that prediabetes is associated with repolarization instability, indicated by elevated values of beat-to-beat short-term QT interval variability, thereby suggesting that an impaired autonomic control precedes the actual onset of diabetes. Last but not least, Altara et al. validated the key importance of targeting microvascular disease, common in both diabetes and obesity, in order to treat heart failure with preserved ejection fraction (HFpEF). Microvascular disease is a growing public health problem, accounting for approximately half of hospital admissions of individuals with heart failure (1, 5, 11, 12).

In summary, the present Research Topic indicates that the exceptional advances achieved in the last decade in understanding the molecular alterations involved in the pathophysiology of both DM and cardiovascular disease are opening new therapeutic opportunities for the treatment of these disorders and, potentially, their future application to the clinical scenario might result to further enhancements in patient care. Furthermore, the exciting findings discussed herein might foster community awareness of these important diseases and stimulate further research in the field.

## Author Contributions

The author confirms being the sole contributor of this work and has approved it for publication.

### Conflict of Interest Statement

The author declares that the research was conducted in the absence of any commercial or financial relationships that could be construed as a potential conflict of interest.
